# Observations on the Prevalence of Fever in Various Parts of the United Kingdom; and on the Eminent Utility of Houses of Recovery, &c.

**Published:** 1819-07-01

**Authors:** 


*Observations on the Prevalence of Fever in various Parts of the United Kingdom; and on the eminent Utility of Houses of Recovery, &c.* By D. J.H. DICKSON, M. D. one of the Physicians to the Clifton Dispensary, &c. Octavo, pp. 34. London, 1819.

Dr. DICKSON's talents, as an able physician and accurate pathologist, are already well known to his professional brethren. In this little pamphlet, distinguished by the classic purity of its style, and the appropriate illustrations of its arguments, our author has embodied and compressed a great mass of evidence, tending to prove, not merely the *utility*, but the absolute *necessity* of Houses of Recovery in all the great towns of the kingdom. Those, therefore, who are about to propose or accelerate such laudable and humane measures, had better possess themselves of the pamphlet, and turn to their advantage the eloquence of our author.

